# Adolescents' Sexual Reproductive Health Service Utilization and Associated Factors Among Bahir Dar City High School Students, Amhara Region, Ethiopia: A Cross-Sectional Study

**DOI:** 10.1155/irem/5367867

**Published:** 2024-12-09

**Authors:** Addis Elefachew, Yibeltal Alemu, Belaynesh Chanie, Eyob Getachew, Solomon Ketema Bogale, Eyob Ketema Bogale

**Affiliations:** ^1^Department of Reproductive Health, Care Ethiopia, Addis Ababa, Ethiopia; ^2^Department of Reproductive Health, School of Medicine and Health Sciences, Bahir Dar University, Bahir Dar, Ethiopia; ^3^Health Promotion and Communication Department, School of Public Health, College of Medicine and Health Sciences, Gondar University, Gondar, Ethiopia; ^4^Department of Nutrition, Antsokiya Gemza Wereda Health Office, North Shoa, Ethiopia; ^5^Department of Health Promotion and Behavioral Sciences, School of Medicine and Health Sciences, Bahir Dar University, Bahir Dar, Ethiopia

## Abstract

**Introduction:** All across the world, youths struggle with a variety of health issues. Adolescents everywhere are entering puberty earlier and engaging in more premarital sex. There was limited evidence about the current adolescent sexual reproductive health (RH) service utilization status and its associated factors in the study area, especially after the occurrence of COVID-19 and the war between the Ethiopian federal government and Tigray regional forces.

**Objective:** The study is aimed at assessing the magnitude of sexual RH service utilization and associated factors among Bahir Dar City high school students, Amhara region, Ethiopia, in 2022.

**Methods:** An institution-based cross-sectional study design was conducted among Bahir Dar City high school students from December 18, 2022, to January 12, 2022. A multistage sampling technique was applied to select study participants. A total of 629 respondents participated in the study. The data were collected using a pretested, structured, self-administered questionnaire. The data were entered into EpiData Version 3.1 and then exported to SPSS Version 25 for analysis. Bivariable and multivariable logistic regression was used for analysis.

**Results:** The magnitude of adolescent sexual RH service utilization was found to be 30.4%. School RH club participation (AOR = 5.93, CI: 3.29–10.71), having ever had sexual exposure (AOR = 6.03, CI: 3.31–10.98), history of sexual RH problems (AOR = 3.26, CI: 1.41–7.51), being perceived as at risk of sexual RH problems (AOR = 6, CI: 3.26–11.04), hearing information about adolescent sexual RH (AOR = 5.19, CI: 3–8.97), and knowing the place to use RH service (AOR = 2.37, CI: 1.47–3.82) were associated with utilization of adolescent sexual RH service.

**Conclusions:** The magnitude of adolescent sexual RH service utilization was found to be 30.4%. School RH club participation, having ever had sexual exposure, a history of sexual RH problems, being perceived as at risk of sexual RH problems, hearing information about sexual RH, and knowing the place where to get RH services were associated with the utilization of sexual RH services.

## 1. Introduction

Adolescents are at higher risk of sexual reproductive health (SRH) issues, including unwanted pregnancy, unsafe abortion, STIs, and sexual violence, due to their increased sexual activity [1]. Young people aged 15–24 make up 15.5% of the global population, with a high proportion in developing countries like Ethiopia accounting for 33% of the total population [2].

The 1994 ICPD in Cairo recognized adolescent-friendly reproductive health (RH) services as an effective strategy for addressing adolescent sexual and reproductive health (ASRH) needs [3], ensuring universal access to quality services without discrimination [4]. Youths worldwide face health issues, including early puberty and premarital sex, but unmet contraceptive needs are twice as high as married women's. Thirty-three percent of new HIV infections occur among youths [5].

In 2016, the prevalence of teenage pregnancy was 19.3% and 21.5% in SSA and East Africa, respectively [6]. Nearly half of the pregnancies among youths aged 15–19 in SSA were unintended [7]. Pregnancy-related deaths are the second-leading cause of death among female youths [8].

SRH disparities exist between developed and developing countries. In developed countries, access to education, healthcare, and contraception leads to better outcomes, but socioeconomic and cultural disparities can still impact SRH outcomes [9, 10]. In developing countries, poor SRH outcomes are often exacerbated by sociocultural barriers, including limited access to sexual education, healthcare services, contraception, early marriage, gender inequality, and inadequate healthcare infrastructure [11, 12, 13].

Youths' access to and use of SRH services is a worldwide concern [10]. Ethiopia offers comprehensive SRH services to youths and adolescents, including information, family planning, condom promotion, pregnancy tests, HIV management, antenatal care, and abortion services [11].

The EDHS 2016 report shows low HIV knowledge among adolescents and youths, particularly rural females, with only 16% having comprehensive knowledge. However, over 90% of urban youths and 69% of rural females know where to get voluntary counseling and testing (VCT) for HIV [14].

In 2018, Ethiopia had 690,000 HIV-positive adults, with 63.88% of them being women and 23,000 were newly infected, leading to 11,000 deaths from AIDS-related illnesses [15]. Adolescent girls and young women aged 15–24 are up to three times more likely to be HIV positive compared to their male counterparts [16].

Adolescent sexual reproductive health rights (ASRHRs) are a key component of some Sustainable Development Goals (SDGs), and these indicators are recommended for Goal 3 on health and Goal 5 on gender equality [12].

Ethiopia has health extension workers providing preventive care and a national adolescent and youth RH strategy, but access to these services is limited. Despite efforts to improve services, high rates of child marriage persist, especially in rural areas [17, 18].

Ethiopia has been enhancing the utilization of ASRH services for underserved adolescent and youth populations since 2018, with collaboration from health partners and the Ethiopian government [19]. The study is aimed at assessing factors associated with the utilization of ASRH services in Ethiopia, following limited evidence post-COVID-19, the war between the government and Tigray, and the sensitive topic of many 12th-grade female students giving birth during university entrance exams.

## 2. Methods and Materials

### 2.1. Study Setting and Period

The study was conducted in Bahir Dar City's secondary and preparatory schools. Bahir Dar is the capital city of Amhara National Regional State; it is situated at a distance of 565 km in the northwest direction from Addis Ababa, the capital city of Ethiopia. There are 11 governmental and 10 private secondary and preparatory schools in Bahir Dar City; five schools were involved in the study. The total number of students enrolled in the high school is 21,867. There are 7763 males and 14,104 females (Bahir Dar City Education Office). Bahir Dar City has two governmental specialized hospitals, one primary governmental hospital, three private primary hospitals, and six health centers. All health facilities are provided by a ASRH service (Bahir Dar City Health Office). The study was conducted from December 18, 2022, to January 12, 2022.

### 2.2. Study Design

An institution-based cross-sectional study was conducted.

### 2.3. Population

#### 2.3.1. Source of Population

All high school students in Bahir Dar City were source of population.

#### 2.3.2. Study Population

The study population consists of randomly selected high school students who were attending classes at the time of data collection in Bahir Dar City.

### 2.4. Eligibility Criteria

#### 2.4.1. Inclusion Criteria

Adolescent high school students who are attending Grade 9, 10, 11, and 12 classes during the data collection period were included. In addition, this high school student, whose age was between 15 and 19 years old during the data collection period and who was willing to participate, was included in the study.

#### 2.4.2. Exclusion Criteria

Students who are seriously ill during the data collection period were excluded.

### 2.5. Sample Size Determination

The sample size was determined using the single population proportion formula with the following assumptions: a 95% confidence level, a margin of error of 5%, a design effect of 1.5, and the magnitude of SRH service utilization of high school students (*p* value = 54.6%) (from the study in the Amhara region, northwest Ethiopia) [20].

After adjustment for 10% nonresponse rate (NR) and 1.5 design effect, the final sample size was 629. The sample size was also calculated using factors associated with ASRH service utilization by considering the following assumptions: two-sided confidence interval (CI) = 95%, power = 80%, ratio (unexposed to exposed) = 1, and 10% NR (Table 1).

Therefore, the sample size obtained by using the single population formula (629) is higher than the sample size calculated by using the second objective (using factors associated with ASRH service utilization). Hence, the minimum sample size to represent the source population was 629.

### 2.6. Sampling Procedure

**Figure 1:**
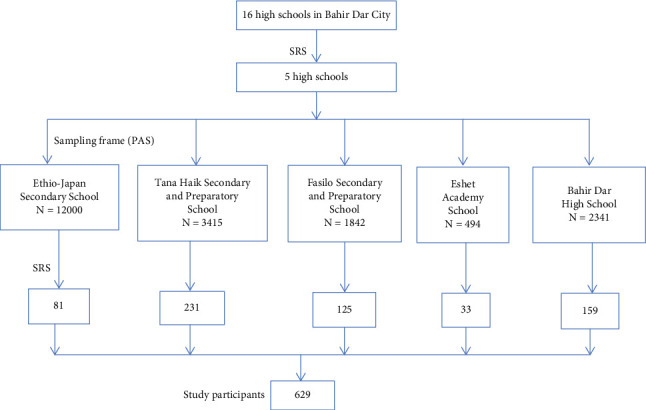
Pictorial presentation of sampling procedure, proportion of allocation, and study participants involved in the selected high schools and preparatory schools at Bahir Dar City, 2022.

A multistage sampling method was applied to select a representative sample of adolescent high school students. In the first stage, high schools in Bahir Dar City were clustered into two groups (regular and irregular), and from each cluster, one school was selected by a simple random sampling method, making a total of two schools. Secondly, high school students were stratified by their grade level of attendance. Then, the sample size was allocated proportionally after obtaining the list of students from the respective school administration. Finally, the study participants were selected from each grade by using simple random sampling from the sampling frame of the student roster (Figure 1).

### 2.7. Dependent Variable

The dependent variable is SRH service utilization (yes or no).

### 2.8. Independent Variable

• Sociodemographic/economic variables: age, sex, grade level, marital status, residence, father's education level, and mother's education level
• Knowledge about ASRH services: exposure to the media, discussion on SRH issues, and awareness of SRHS
• Behavioral-related variables: participating in a school club, having sexual friends, having previous SRH-related problems

### 2.9. Operational Definition

#### 2.9.1. Reproductive Health Service Utilization

This is the utilization of one of the SRH services (family planning, abortion services, STI or HIV testing and treatment, awareness of SRH services, and discussions on SRH service utilization) in the previous year [21].

#### 2.9.2. Knowledge of ASRH Service

If the respondents mentioned at least five ASRH services on their own, they were considered to have good knowledge; otherwise, they were considered to have poor knowledge [20].

### 2.10. Data Collection Tools and Methods

Literature is reviewed to develop a questionnaire [22]. The data were collected using a pretested, structured, self-administered questionnaire. The questionnaire was prepared first in English and then translated to the local language of Amharic and translated back to English by a third person who was native to Amharic and had experience in translation. Questionnaires are categorized into sociodemography, exposure to ASRH information, ASRH service utilization, and ASRH service availability knowledge. Four health extension workers collected the data. One health officer was supervising the data collection. Each day, questionnaires were checked for completeness and consistency by the principal investigator and supervisor.

### 2.11. Data Quality Control

A pretest was done on 5% of the sample size before the actual data collection in another unselected high school, Eshet Senior Secondary School, in Bahir Dar City, in order to ensure that respondents were able to understand the questions, check the wording and logic, and skip the order of the questions in a sensible way. Amendments were made to the questions after pretesting. The questionnaire was prepared first in English and then translated to the local language of Amharic and translated back to English by a third person who was native to Amharic and had experience in translation. The validity of the tools was assessed by experts. The reliability test was checked using Cranach's alpha of 0.7 as a cut-off point, and multicollinearity between independent variables was also checked.

### 2.12. Data Processing and Analysis

Data were cleaned, coded, and entered into EpiData Version 3.1 and exported to Statistical Package for Social Science (SPSS) Version 25 software for further analysis. Descriptive statistics were used to describe the distributions of the variables. A binary logistic analysis was carried out to see the association between the dependent variables and each independent variable. In bivariate analysis, variables whose *p* value was ≤ 0.25 were used to select candidate variables for multivariable analysis.

In the final model, a *p* value < 0.05 was considered statistically significant. The goodness of fit of the final model was checked using the Hosmer and Lemeshow test of a good fit with a *p* value > 0.05. The odds ratio was used to observe the strength of the association between a dependent variable and each significant independent variable.

### 2.13. Ethical Considerations

Ethical approval for this study was obtained from the GAMBY Medical and Business College with Reference Number 109/2023, and letters of cooperation were received from the Amhara Regional Educational Office. Written informed consent was obtained from each study participant prior to the commencement of data collection.

Permission to undertake the study was obtained at all levels. For participants whose ages were less than or equal to 18 years old, the school principal and teachers were given detailed information about the purpose of the study; data collection procedures; and possible risks, discomforts, and benefits of participating in the study. Considering ASRH is a sensitive issue within society, study participants < 18 years old were asked for consent themselves. Written informed consent was obtained from all students > 18 years old who participated in the study, and their decision not to participate in the study was respected. The participants were allowed to consider their participation and were given the opportunity to withdraw from the study if they wished to do so.

## 3. Results

### 3.1. Sociodemographic Characteristics of Respondents

A total of 629 high school students were included in the study, with a response rate of 96.34% and a 3.7% NR due to incomplete responses. The mean age of the study participants was 17 years old, with a standard deviation (SD) of 1.00577 years, and the most frequent age category was 15–17 years old. Almost all 568 (93.7%) of the respondents were single. The majority (488 or 80.5%) of the respondents were Orthodox Christian followers (Table 2).

### 3.2. Sex-Related Characteristics, Source of Information, and Knowledge of Study Participants

Two hundred six (34%) of the participants ever had a boy or girlfriend, and 188 (31%) of the participants were sexually active. The majority (568, 93.73%) of the participating high school students and adolescents had been exposed to SRH information. Regarding the discussion of SRH issues, nearly half (47.2%) of the respondents have ever discussed SRH issues. Of the total participants, about 237 (39.1%) knew of health facilities that provide SRH services (Table 3).

### 3.3. Magnitude of ASRH Service Utilization

**Figure 2:**
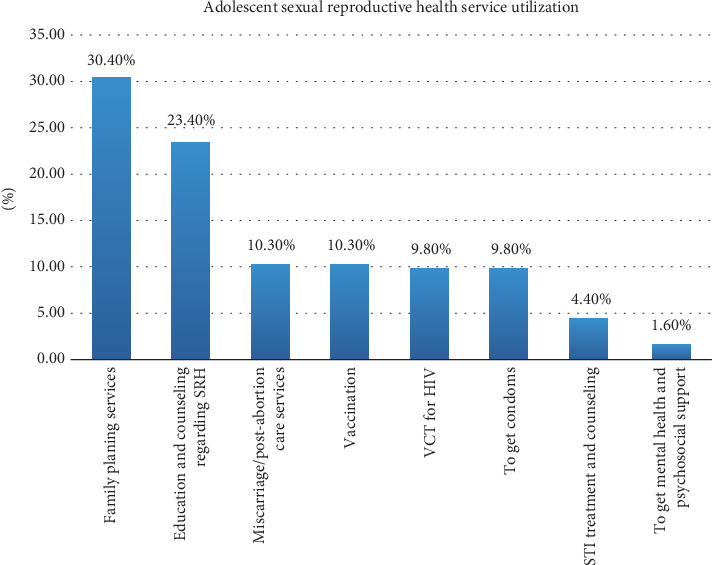
Utilization of ASRH services among high school students in Bahir Dar City, northwest Ethiopia.

The magnitude of ASRH service utilization was found to be 30.4% (184) with a 95% CI of 26.7 and 34.2. The most frequently used components of ASRH services were family planning services, education, and counseling regarding ASRH, followed by miscarriage and postabortion care services, vaccination, getting a condom, VCT for HIV, STI treatment and counseling, and getting mental health and psychosocial support (Figure 2).

### 3.4. Factors Associated With Utilization of ASRH Service

In bivariable analysis, the age of the respondent, marital status, educational status of the father, educational status of the mother, living arrangements, having a boy or girlfriend, occupation of the father, occupation of the mother, source of pocket money, knowledge, having ever had sexual exposure, school club participation, history of SRH problems, being perceived as at risk of SRH problems, hearing information about ASRH, and knowing the place to get the service were found to be candidate variables for multivariable analysis at *p* value less than or equal to 0.25 (Table 4).

On multivariable analysis, sexual exposure, school club participation, history of SRH problems, perceived risk of SRH problems, hearing information about ASRH, and knowing the place where to get the RH service were significantly associated with utilization of the ASRH service at a *p* value less than 0.05 (Table 4).

The odds of ASRH service utilization among high school students who had a history of sexual intercourse were 6.03 times higher than those among their counterparts (AOR = 6.03; CI: 3.31–10.98). The odds of ASRH service utilization among high school students who participated in the RH school club were 5.93 times higher than those among their counterparts (AOR = 5.93, CI: 3.29–10.71). The odds of ASRH service utilization among high school students who perceived they were at risk of SRH problems were six times higher than those among their counterparts (AOR = 6, CI: 3.26–11.04). The odds of ASRH service utilization among high school students who knew the place to get adolescent RH services were 2.37 times higher than those among their counterparts (AOR = 2.37, CI: 1.47–3.82). The odds of ASRH service utilization among high school students who heard about ASRH were 2.79 times higher than those among their counterparts (AOR = 5.19, CI: 3–8.97). The odds of ASRH service utilization among high school students who had a history of SRH problems were 1.6 times higher than those among their counterparts (AOR = 3.26, CI: 1.41–7.51) (Table 4).

## 4. Discussion

The results of the study revealed that the proportion of high school students who utilized ASRH services was found to be 30.4% (184) with a 95% CI (26.7, 34.2). This finding was higher than the study findings from Mecha district (8.4%) [23], Machakel district (21.5%), east Gojjam zone [24], Mekele (22%) [25], western Ethiopia (8.6%) [26], South Gondar (24.6%), Wereta Ethiopia [27], Dawuro zone (26%), southwest Ethiopia [28], Haramaya district (23.5%) [29], and Nekemte town (21.2%) in Oromia region [30]. This difference might be due to the presence and exertion of nongovernmental organizations (NGOs) like the Family Guidance Association (FGA) in Ethiopia, which have increased their contribution to the expansion of adolescent and youth sexual and reproductive service provision in the study area.

This might significantly alter how often ASRH services are used compared to the earlier findings. Besides this, the result of this study was higher compared to that of other studies done in urban Nepal (9.2%) [31] and Makassar, Indonesia [32]. This discrepancy might be due to the difference in sociodemographic characteristics of participants and the study settings in terms of the availability and accessibility of ASRH service facilities. Also, the study in Nepal's Bhaktapur district found that, during the survey, youth-friendly health services were not properly integrated into urban clinics. This may also be the reason for the lower utilization of ASRH services.

On the other hand, this finding was lower than the study findings of 48.9% in Addis Ababa [31] and 54.6% in the Amhara region, northwest Ethiopia [20]. This difference might be due to age and maturity differences. For instance, the study in Addis Ababa was carried out among undergraduate students at Addis Ababa University, whose age and maturity are substantially higher than those of the study participants. University students may also have a high level of peer influence and a high sense of risk despite being generally free from family and community factors. All of these could have affected how people used SRH services. The majority of the out-of-school adolescents studied in the Amhara area of northwest Ethiopia may have had sexual exposure and may be married.

The results of this study indicate a lower prevalence compared to previous research, including rates of 32.2% in Bahir Dar [34] and 32.7% (ranging from 29.0% to 36.6%) in the North Shewa Zone [35]. Additionally, the findings are notably lower than those reported in Enugu State, Nigeria (86.7%) [36], and Ghana (55.8%) [37]. This disparity may stem from various factors, such as sociocultural barriers, perceptions among adolescents (including concerns about visibility when accessing SRH services), and challenges related to service delivery (such as provider attitudes, accessibility, and service hours).

In this study, students who had ever had sexual exposure were 6.03 times more likely to utilize the ASRH service compared with those who had not. This finding is in line with the studies conducted in Mecha district [23], western Ethiopia [26], and North Shewa [35]. These similarities might be due to the difference in risk perception. Teenagers who engage in sexual activity may perceive unintended pregnancy, abortion, HIV, and other STIs as being more dangerous than those who refrain from doing so [7, 9]. So, people might prepare and employ defensive strategies when engaging in sexual practice.

This finding showed a significant association between prior sexual exposure and the utilization of ASRH services among students. The observed trend aligns with similar studies conducted in Mecha district [23], western Ethiopia [26], and North Shewa [35], indicating a consistent pattern across different regions. This suggests that individuals with prior sexual exposure may have a heightened awareness of their RH needs, leading to a greater propensity to seek ASRH services.

In this study, the odds of ASRH service utilization were 5.93 times higher among students who participated in the RH school club compared to those who did not participate in the RH school club. This finding was in agreement with the study findings in Mecha district [23]. These similarities may be caused by the fact that students who participate in the RH school club frequently talk about issues related to SRH, obstacles to using services, and risks related to SRH; develop life skills; share experiences; and are aware of these issues. As a result, this may lead to higher ASRH service utilization.

In this study, perceived risk of SRH problems showed a significant association with ASRH service utilization. Students who perceived they were at risk of SRH problems were six times more likely to utilize the ASRH service compared with their counterpart. This might be because when adolescents perceive that they are at risk of getting SRH problems, they are more likely to utilize ASRH service like VCT.

This study also indicated that students who knew about adolescent RH services were significantly associated with ASRH service utilization. High school students who knew about adolescent RH services were about 2.37 times more likely to utilize ASRH services compared with those who did not know how to get adolescent RH services. This finding was in agreement with the study findings in Haramaya district [29]. This could be because students who know where to go to use ASRH services might have a greater possibility of utilizing ASRH services.

In this study, the odds of ASRH service utilization were 2.79 times higher among students who heard about ASRH compared with those who did not hear about ASRH. This finding was in agreement with the study findings in western Ethiopia [26], Dawuro zone [28], and Haramaya district [29]. This could be due to having a source of information that may improve adolescents' awareness of SRH-related issues and their health-seeking behavior.

This study also indicated that students who had a history of SRH problems were significantly associated with ASRH service utilization. High school students who had a history of SRH problems were 1.6 times more likely to utilize ASRH services compared with their counterparts. This finding was consistent with the study findings in Bahir Dar [34] and North Shewa Zone [35]. This could be because having RH problems like unwanted pregnancy, STIs, and HIV pushes adolescents to seek treatment for their illnesses, which increases ASRH service utilization.

The limitations of this study include its reliance on self-reported data, which may be subject to recall bias or social desirability bias. Additionally, the study focused exclusively on high school students, which may not fully represent the broader adolescent population. Furthermore, the study's cross-sectional design limits its ability to establish causality or determine long-term trends in ASRH service utilization. This study also did not address some behavioral variables such as chewing khat, drinking alcohol, and other substance abuse, so we recommend future researchers address them. Finally, while efforts were made to ensure the validity and reliability of the findings, the study's reliance on a single data collection method may have introduced methodological limitations.

The study's findings carry weighty implications. They shed light on the factors affecting the utilization of ASRH services among high school students, guiding focused interventions to enhance access. Pinpointing factors such as the involvement of NGOs enables policymakers to customize initiatives to match specific requirements, emphasizing the necessity of incorporating youth-friendly health services. Ultimately, this understanding can lead to the development of more efficient approaches for advancing ASRH.

These findings guide policymakers in crafting effective policies on ASRH. By understanding factors influencing service utilization, policymakers can prioritize partnerships with NGOs and integrate youth-friendly services, leading to improved access and outcomes for adolescents.

## 5. Conclusion

This study revealed that the overall magnitude of ASRH service utilization was found to be low. Reproductive health school club participation was significantly associated with sexual exposure; a history of SRH problems, being perceived as at risk of SRH problems, hearing information about ASRH, and knowing the place to get the service were significantly associated with utilization of ASRH. The purpose of this study was to assess SRH service utilization and associated factors among high school students. Hence, it is important to promote adolescent utilization of SRH services by increasing their source of information about ASRH, their perception of the risk of getting SRH problems, and their participation in the RH school club.

## Figures and Tables

**Table 1:** Sample size for associated factors using Epi Info 7.

**Factors**	**%outcome in unexposed**	**%outcome in exposed**	**Power**	**Confidence level**	**Odds ratio**	**Calculated sample size**	**S** **a** **m** **p** **l** **e** **size** + **NR**	**Reference**
Participated in the reproductive health school club	14.7%	23.3%	80%	95%	2.36	268	294	[23]
Having sexual exposure	11.6%	54.4%	80%	95%	11.27	32	36	[23]
Discussed with their families SRH issues	15.1%	25.4%	80%	95%	1.43	474	521	[23]
Personal income or pocket money	16.8	21	80%	95%	3.6	104	114	[23]

**Table 2:** Sociodemographic characteristics of high school students in Bahir Dar City, northwest Ethiopia, 2023 (*n* = 606, nonresponse 23 students).

**Characteristics**	**Frequency**	**Percent**
Age of student (in years)		
18–19	186	30.7
15–17	420	69.3
Sex		
Male	216	35.6
Female	390	64.4
Marital status		
Single	568	93.7
Married	38	6.2
Religion		
Orthodox	488	80.5
Muslim	68	11.2
Protestant	50	8.3
Educational status		
Grade 9	248	40.9
Grade 10	198	32.7
Grade 11	112	18.5
Grade 12	48	7.9
Educational program		
Day	480	79.2
Night	126	20.8
Educational status of father		
Unable to read and write	162	26.7
Primary	129	21.3
Secondary	125	20.6
College and above	190	31.4
Educational status of mother		
Unable to read and write	188	31.0
Primary	152	25.1
Secondary	160	26.4
College and above	106	17.5
Living arrangements		
Father only/mother only	64	10.6
Living with others (spouse, relatives, friends/peers/alone)	178	29.4
Living with both parents	364	60.1
Occupational status of mothers		
Housewife	384	63.4
Farmer	60	9.9
Government employee	78	12.9
Others (merchant and self-employee)	84	13.9
Occupational status of fathers		
Farmer	204	33.7
Merchant	120	19.8
Government employee	218	36.0
Self-employee	64	10.6
Do you have pocket money		
Yes	71	11.7
No	535	88.3

**Table 3:** Behavior-related characteristics, source of information, and knowledge of high school students in Bahir Dar City, northwest Ethiopia, 2023 (*n* = 606).

**Characteristics**	**Frequency**	**Percent**
Do you have a boy/girlfriend		
Yes	206	34.0
No	400	66.0
Ever had sexual intercourse		
Yes	188	31.0
No	418	69.0
Do you have source of information about SRH issue		
Yes	568	93.73
No	38	6.27
Have you ever discussed about SRH issue		
Yes	292	48.2
No	314	51.8
Do you think you are at risk of SRH problems (HIV, pregnancy, sexually transmitted infections, early parenthood, abortion…)		
Yes	207	34.2
No	399	65.8
Do you participate in SRH school club		
Yes	131	21.6
No	475	78.4
Have you ever had SRH-related problem (HIV, pregnancy, sexually transmitted infections, early parenthood, abortion)		
Yes	58	9.6
No	548	90.4
Know SRH facility whereabout		
Yes	237	39.1
No	369	60.9

Abbreviations: HIV, human immunodeficiency virus; SRH, sexual reproductive health.

**Table 4:** Bivariate and multivariable association of utilization of ASRH services and independent factors among high school students in Bahir Dar City, northwest Ethiopia, 2023 (*n* = 606).

**Variable**	**Category**	**ASRH service utilization**		**COR (95%)**	**AOR (95%)**	**p** ** value**
		**Yes**	**No**			
Age	18–19	136	284	1.37 (0.95–2.05)	1.21 (0.39–3.71)	0.74
	15–17	48	138	1	1	

Marital status	Married	18	14	3.16 (0.78–4.16)	1.37 (0.89–2.13)	0.15
	Single	166	408	1	1	

Educational status	Grade 10	61	137	0.92 (2.71–4.17)	2.97 (3.46–6.02)	0.3
	Grade 11	22	90	0.50 (2.05–5.41)	3.36 (1.64–6.88)	0.1
	Grade 12	20	28	1.47 (1.92–2.76)	4.67 (2.06–10.59)	0.81
	Grade 9	81	167	1	1	

School club	**Yes**	**64**	**254**	**0.35 (0.24**–**6.01)**	**5.93 (3.29**–**10.71)**	**0.001**
	No	120	168	1	1	

At risk of SRH problems	**Yes**	**34**	**173**	**0.32 (0.22**–**6.03)**	**6 (3.26**–**11.04)**	**0.045**
	No	150	249	1	1	

Do you have a boy/girlfriend	Yes	86	120	2.2 (0.44–4.01)	0.19 (0.33–0.12)	0.09
	No	98	302	1	1	

Sexual exposure	**Yes**	**41**	**147**	**0.53 (0.48**–**2.04)**	**6.03 (3.31**–**10.98)**	**0.001**
	No	143	275	1	1	

History of RH problems	**Yes**	**33**	**109**	**0.62 (0.34**–**4.94)**	**3.26 (1.41**–**7.51)**	**0.001**
	No	151	313	1	1	

Living arrangements	Father only/mother only	26	38	1.73 (0.34–1.02)	0.46 (0.12–1.72)	0.249
	Living with others (spouse, relatives, friends/peers/alone)	55	123	1.13 (0.62–1.35)	0.10 (0.02–0.070)	0.927
	Living with both parents	103	261	1	1	

Education of mother	Primary	44	108	0.79 (1.52–2.43)	1.29 (0.73–2.32)	0.57
	Secondary	49	111	0.86 (2.41–5.76)	1.19 (0.66–2.12)	0.64
	College and above	27	79	0.66 (1.72–3.54)	1.27 (0.69–2.29)	0.38
	Unable to read and write	64	124	1	1	

Occupational status of father	Farmer	68	136	1.63 (1.91–3.01)	0.524 (0.234–1.17)	0.116
	Merchant	36	84	1.4 (2.12–5.74)	0.68 (0.29–1.59)	0.0369
	Government employee	65	153	1.39 (1.85–3.21)	0.69 (0.31–1.53)	0.0.361
	Self-employee	15	49	1	1	

Occupational status of mother	Farmer	14	46	0.62 (0.38–1.15)	0.30 (0.08–2.42)	0.26
	Government employee	19	59	0.66 (0.36–1.44)	0.33 (0.04–2.86)	0.32
	Others (merchant and self-employee)	26	58	0.92 (0.48–2.33)	0.38 (0.04–3.3)	0.38
	Housewife	126	258	1	1	

Occupation of mother	No	157	378	0.9 (0.91–5.01)	0.79 (0.38–1.64)	0.53
	Yes	27	44	1	1	

Heard information about ASRH	**Yes**	**116**	**306**	**0.64 (0.08**–**2.91)**	**5.19 (3.0**–**8.97)**	**0.001**
	No	68	116	1	1	

Know service whereabout	**Yes**	**88**	**250**	**0.63 (1.40**–**3.57)**	**2.37 (1.47**–**3.82)**	**0.03**
	No	96	172	1	1	

Knowledge	Knowledgeable	30	48	1.51 (1.02–2.43)	0.43 (0.22–0.82)	0.11
	Not knowledgeable	154	374	1	1	

*Note*: Bold cell body entries indicate value for only variables significantly associated with the dependent variable.

## Data Availability

The authors have nothing to report.

## Nomenclature

ETB: Ethiopian birr
FV: fruit and vegetables
NCD: nonchronic disease
SRS: systematic random sampling
SPSS: Statistical Package for Social Science
WHO: World Health Organization

## References

[1] Okeke S. R., Idriss-Wheeler D., Yaya S. (2022). Adolescent pregnancy in the time of COVID-19: What are the implications for sexual and reproductive health and rights globally?. *Reproductive Health*.

[2] Federal Democratic Republic of Ethiopia Ministry of Health (2021). *National adolescent and youth health strategy (2021-2025)*.

[3] World Health Organization (1999). *Programming for Adolescent Health and Development: Report of WHO/UNFPA/UNICEF Study Group on Programming for Adolescents Health*.

[4] OECD (2012). *Tackling the root causes of gender inequalities in the post-2015 development agenda, submission to the global thematic consultation on addressing inequalities*.

[5] UNAIDS (2018). *Joint United Nations Programme on HIV/AIDS— State of the epidemic*.

[6] Kassa G. M., Arowojolu A. O., Odukogbe A. A., Yalew A. W. (2018). Prevalence and determinants of adolescent pregnancy in Africa: a systematic review and meta-analysis. *Reproductive Health*.

[7] Mittiku Y. M., Tamiru A. T., Rade B. K. (2021). The significant influence of history of induced abortion on the utilization of long-acting reversible contraceptive methods in the immediate post-abortion period, Northern Ethiopia. *The European Journal of Contraception & Reproductive Health Care*.

[8] Alderman E. M., Breuner C. C. (2019). Unique needs of the adolescent. *Pediatrics*.

[9] Liang Y., Hee J., Peng C., Li C., Cao W., Tang K. (2022). Comparing access to sexual and reproductive health services among sexual minority youths and their peers: findings from a national survey in China. *BMC Public Health*.

[10] Kemp J., Maclean G. D., Moyo N. (2021). *Global Midwifery: Principles, Policy, and Practice*.

[11] Human Development Resource Centre (2011). *Adolescent reproductive health in Ethiopia*.

[12] MOHE (2021). *Reproductive Health Strategic Plan 2021-2025*.

[13] Ethiopia Demographic and Health Survey (EDHS) (2016). *Key Indicators Report. The DHS Program*.

[14] Dingeta T., Oljira L., Worku A., Berhane Y. (2019). Unmet need for contraception among young married women in eastern Ethiopia. *Journal of Contraception*.

[15] West C. A., Chang G. C., Currie D. W. (2021). Unawareness of HIV infection among men aged 15–59 years in 13 sub-Saharan African countries: findings from the population-based HIV impact assessments, 2015–2019. *JAIDS Journal of Acquired Immune Deficiency Syndromes*.

[16] Comins C. A., Rucinski K. B., Baral S., Abebe S. A., Mulu A., Schwartz S. R. (2020). Vulnerability profiles and prevalence of HIV and other sexually transmitted infections among adolescent girls and young women in Ethiopia: a latent class analysis. *PLoS One*.

[17] Jones N., Presler-Marshall E., Hicks J., Baird S., Chuta N., Gezahegne K. (2019). *Adolescent Health, Nutrition, and Sexual and Reproductive Health in Ethiopia, A Report on GAGE Ethiopia Baseline Findings*.

[18] Jain A., Ismail H., Tobey E., Erulkar A. (2017). *Understanding Adolescent and Youth Sexual and Reproductive Health-Seeking Behaviors in Ethiopia: implications for Youth Friendly Service Programming, Research Report*.

[19] Africa A. H. (2018). *Creating lasting health change in Africa (Annual Report)*.

[20] Liyeh T. M., Goshu Y. A., Belay H. G., Tasew H. A., Mihiretie G. N., Ayalew A. B. (2021). Youth reproductive health service utilization and associated factors among Amhara region female night students, Ethiopia. *BioMed Research International*.

[21] Regmi P. R., Van Teijlingen E., Simkhada P., Acharya D. R. (2010). Barriers to sexual health services for young people in Nepal. *Journal of Health, Population, and Nutrition*.

[22] UNFPA (2009). *Adolescent Sexual and Reproductive Health Toolkit for Humanitarian Settings*.

[23] Alehegn B. G., Mulunesh T. T., Yilkal T. A., Abebaw A. G. (2018). Sexual and reproductive health services utilization and associated factors among preparatory school students in Mecha district, northwest Ethiopia: cross sectional study. *Journal of Gynecology and Women's Health*.

[24] Abajobir A. A., Seme A. (2014). Reproductive health knowledge and services utilization among rural adolescents in east Gojjam zone, Ethiopia: a community-based cross-sectional study. *BMC Health Services Research*.

[25] Bilal S. M., Spigt M., Dinant G. J., Blanco R. (2015). Utilization of sexual and reproductive health services in Ethiopia – does it affect sexual activity among high school students?. *Sexual & Reproductive Healthcare*.

[26] Tilahun T., Bekuma T. T., Getachew M., Seme A. (2021). Assessment of access and utilization of adolescent and youth sexual and reproductive health services in western Ethiopia. *Reproductive Health*.

[27] Abate A. T., Ayisa A. A., W/Mariam T. G./. M. (2019). Reproductive health services utilization and its associated factors among secondary school youths in Woreta town, South Gondar, North West Ethiopia: a cross sectional study. *BMC Research Notes*.

[28] Yonas F., Chernet A. A. (2022). Sexual and reproductive health service utilization, and associated factors among high school learners in the Dawuro zone, Southwest Ethiopia. *African Journal of Reproductive Health*.

[29] Abdurahman C., Oljira L., Hailu S., Mengesha M. M. (2022). Sexual and reproductive health services utilization and associated factors among adolescents attending secondary schools. *Reproductive Health*.

[30] Binu W., Marama T., Gerbaba M., Sinaga M. (2018). Sexual and reproductive health services utilization and associated factors among secondary school students in Nekemte town, Ethiopia. *Reproductive Health*.

[31] Kiran Bam F. H., Rajendra Kumar B. C., Sophia Newman M., Chaudhary A. H., Thapa R., Bhuyia I. (2015). Perceived sexual and reproductive health needs and service utilization among higher secondary school students in urban Nepal. *American Journal of Public Health Research*.

[32] Violita F., Hadi E. N. (2019). Determinants of adolescent reproductive health service utilization by senior high school students in Makassar, Indonesia. *BMC Public Health*.

[33] Mekbib B., Demissei D. B. (2023). Sexual reproductive health service utilization and associated factors among undergraduate students of Addis Ababa University in Ethiopia. *Journal of Health, Population, and Nutrition*.

[34] Abebe M., Awoke W. (2014). Utilization of youth reproductive health services and associated factors among high school students in Bahir Dar, Amhara regional state, Ethiopia. *Journal of Epidemiology*.

[35] Demeke F., Yohannes T., Abera N., Belayneh F., Nigussie S. (2022). Youth friendly services utilization and associated factors among school youths in North Shewa Zone, Amhara Region, Ethiopia: a mixed-method study. *SAGE Open Medicine*.

[36] Odo A. N., Samuel E. S., Nwagu E. N., Nnamani P. O., Atama C. S. (2018). Sexual, and reproductive health services (SRHS) for adolescents in Enugu state, Nigeria: a mixed methods approach. *BMC Health Services Research*.

[37] Appiah S. C., Badu E., Dapaah J., Takyi H., Abubakari M. (2015). Youth friendliness of sexual and reproductive health service delivery and service utilization in the Kwadaso Sub-Metro of the Ashanti Region, Ghana. *International Journal of Innovation and Applied Studies*.

